# In reply: Comparing performance of McGrath MAC videolaryngoscope in morbidly obese and nonobese

**DOI:** 10.3906/sag-2003-230

**Published:** 2020-12-17

**Authors:** Zehra İpek ARSLAN

**Affiliations:** 1 Department of Anesthesiology and Reanimation, Faculty of Medicine, Kocaeli University, Kocaeli Turkey

****To the Editor,****

First of all, I would like to thank the Editor for giving me the chance to reply in this ‘Letter to the Editor’ regarding our newly published article called ‘Tracheal intubation with the McGrath MAC X-Blade videolaryngoscope in morbidly obese and nonobese patients’[1]. Additionally, I am also grateful to the authors for their didactic diagnosis and for having given priority to the airway management of an obese patient consistent with our manner as well. This is an opportunity for us to discuss and improve our knowledge together. I am truly enthusiastic about the insight and I learn while replying to their assignments.

We divided the patients into 2 groups, nonobese (Body Mass Index (BMI) < 30) and morbidly obese (BMI > 35) as many prior studies have done as noted in their literature: normal; BMI 20 to 25 kg.m–2, overweight; BMI 25 to 30 kg.m–2, obesity; BMI > 30 kg.m–2, morbid obesity; BMI > 40 kg.m–2 [2]. Indeed, as mentioned in our study, the nonobese group (BMI < 30) could be identified as the [normal (BMI between 18.5–24.9) and the overweight (BMI 25.0–29.9)] group. The morbidly obese group (BMI > 35) was also be referred to [Class II and Class III obesity Group] according to the new World Health Organization (WHO) obesity classification: normal; BMI 18.5 to 24.9 kg.m–2, overweight; BMI 25 to 29.9 kg.m–2, Class I obese; 30 to 34.9 kg.m–2, Class II obese; 35 to 39.9 kg.m–2, Class III obese; BMI > 40 kg.m–2 [3]. We will use this classification in our future trials. I agree with the authors that we have to note that a Stratified random sampling was used for grouping according to BMI in this trial. Enrollment of these patients was conducted between 1 August 2018 to 1 December 2018 and the patients meeting the enrollment criteria provided written consent for this study. I certainly agree with the authors that preoxygenation in the ramped position (semisitting position) is the ideal and recommended position for a morbidly obese patient. Intubation in the ramped position is a continuing discussion among experts in the field and still an ongoing debate to be explored [4]. We need future trials comparing the intubation of a patient placed in the ramped position and in a supine position. Also, there was a published multicenter trial in critically ill adults published in 2017 which demonstrated that the ramped position did not improve oxygenation during intubation and it worsened the glottis view and increased the number of intubation attempts [5]. In our daily practice we preoxygenate our obese patients in the ramped position and during awake videolaryngoscopic or fiberoptic intubation. However, in anesthetized obese patients we intubate in the supine position. In our trial, we wanted to demonstrate helpful maneuvers while directing the tube into the trachea during intubation with a videolaryngoscope, without the use of a stylet. Even while using a stylet we could require these maneuvers to direct the tube into the trachea and the Cochrane database showed that using a stylet did not improve intubation [6]. In addition, as we all know that use of a stylet will cause harm to our patient. Cricoid pressure improved the Cormack-Lehane grades of some videolaryngoscopes, some did not. It depends on the type of the videolaryngoscope. Cricoid pressure improves the laryngoscopic view and eases the tube insertion during intubation with the McGrath MAC [7].

Our sample size is not calculated for postoperative minor complications. If the sample size increased, the results would be different. We mentioned this fact as a limitation of our study in the discussion section. We did not use any scale to assess the severity of sore throat between the groups. It would be more revealing if we evaluate the level of sore throat according to a specific scale.
